# Sports Nutrition Knowledge among Mid-Major Division I University Student-Athletes

**DOI:** 10.1155/2016/3172460

**Published:** 2016-10-31

**Authors:** Ashley Andrews, Janet R. Wojcik, Joni M. Boyd, Charles J. Bowers

**Affiliations:** Department of Physical Education, Sport and Human Performance, Winthrop University, Rock Hill, SC 29733, USA

## Abstract

Competitive athletes have goals to optimize performance and to maintain healthy body composition. Sports nutrition is a component of training programs often overlooked by student-athletes and their coaches. The purpose of this study was to examine student-athletes' sports nutrition knowledge across sex, class level, team, and completion of prior nutrition coursework. Participants included 123 mid-major Division I university student-athletes (47 females and 76 males) from baseball, softball, men's soccer, track and field, and tennis. The student-athletes completed a survey questionnaire to determine adequate sports nutrition knowledge (mean ≥ 75%). The overall mean sports nutrition knowledge score for the student-athletes was 56.9% which was considered inadequate sports nutrition knowledge (mean < 75%). Only 12 student-athletes achieved adequate sports nutrition knowledge score of 75% or higher. There were no differences by sex, class level, team, and completion of prior nutrition coursework. Student-athletes' inadequate sports nutrition knowledge may place them at nutrition risk, lead to impaired performance, and affect their lean body mass and energy levels. Athletics personnel should not assume student-athletes have adequate sports nutrition knowledge. Athletic departments may make available a board certified Sports Dietitian or Registered Dietitian and offer classroom or online courses facilitating student-athletes to optimize nutrition knowledge and behaviors.

## 1. Introduction

Student-athletes have to excel in the classroom and on the playing field. Improving their skills during hours of sport-specific practice is only the beginning of the training regimen that is necessary to be a successful student-athlete. Additional aspects of a student-athlete's training regimen should include proper sports nutrition and effective strength training. These two aspects can be overlooked, but they are extremely important factors student-athletes should incorporate into their training plans.

Typically, competitive athletes have two straightforward dietary goals which include eating to maximize performance and eating to obtain optimal body composition. No specific eating regimen will directly increase strength, aerobic endurance, or power, but an adequate nutrient dense diet will allow athletes to train and compete to the best of their abilities [1]. The demands student-athletes place upon their bodies during sport-specific and non-sport-specific training need to be balanced with proper nutrition, especially for females in endurance sports who could be at risk of female athlete triad [2]. The importance of eating a nutrient dense diet is often times not emphasized enough to student-athletes by the professional staff who have the most contact with them: team coaches, strength and conditioning specialists, and athletic trainers. Although some larger universities may employ a full-time or part-time Registered Dietitian (RD) or Board Certified Specialist in Sports Dietitian (CSSD), these professionals are only available at approximately 10% of collegiate athletic departments [3]. There is no comprehensive nutrition education program for athletes mandated by the National Collegiate Athletic Association (NCAA) similar to other required academic support and life skills programs [3, 4].

Optimal nutrition facilitates and enhances physical activity, athletic performance, and recovery [5]. Student-athletes need to develop healthy eating habits in order to maintain body weight and health while maximizing training effects. Athletes may not even have knowledge of basic nutrition concepts such that fruits and vegetables are good sources of carbohydrates in addition to grains [4]. As student-athletes enter the collegiate setting, coaches, strength and conditioning specialists, and athletic trainers may wish to assess the student-athlete's knowledge about sports nutrition if a Dietitian is not available. Obtaining this information will allow these professionals to focus on areas which need improvement and potentially make referrals. Due to the demands placed on the student-athletes in sport-specific and non-sport-specific training, education about how to achieve an optimal energy balance is key in order for the student-athletes to perform at their highest level [5].

Student-athletes often rely on team coaches, strength and conditioning specialists, and athletics trainers for sports nutrition knowledge. However, studies have examined the nutrition knowledge of these professionals that indicated they may not be the best source for the student-athletes [3, 6, 7]. Torres-McGehee et al. studied 579 student-athletes, coaches, athletic trainers, and strength and conditioning coaches to examine sports nutrition knowledge on a questionnaire they developed. Adequate sports nutrition knowledge was determined if the participant obtained an overall score of at least 75% in all domains. Athletic trainers and strength and conditioning specialists had the highest average scores of 77.8% and 81.6%, respectively, which were higher than the team coaches and the student-athletes [6]. Rockwell et al. assessed the nutrition knowledge, opinions, and practices of 53 coaches, strength and conditioning specialists, and athletic trainers at a Division I university. Strength and conditioning specialists scored significantly more correct answers at 80% compared to team coaches at 62% and athletic trainers at 66%. Coaches who worked with female athletes or both male and female athletes and coaches with at least 15 years' experience gave more correct responses. Not only should the student-athletes be tested on nutrition knowledge and behaviors, but the coaching staff and other professionals who work closely with them should also be studied to ensure the student-athletes are receiving correct and current sports nutrition education, especially if there is no Dietitian [7].

There is a history of student-athletes reporting low sports nutrition knowledge, though findings are mixed due to differences in populations and instrumentation [8]. As far back as 1992, Jacobson and Aldana found only 26.7% of 812 Division I student-athletes could correctly identify fat-soluble vitamins and 50% knew correct functions of protein, but 85% could identify carbohydrates as an immediate energy source. These participants indicated their sources of nutrition knowledge were magazines, athletic trainers, friends, and coursework [9]. Cultural and religious factors may also influence student-athlete nutrition [8]. The Internet can also be a reference for sports nutrition knowledge [8, 10] but may contain inaccurate information from questionable sources. A follow-up to the 2001 study [9] with 330 student-athletes found lower sports nutrition knowledge in that only 29% could identify correct carbohydrate recommendations while even fewer could correctly identify fat and protein recommendations. Female student-athletes reported receiving their sports nutrition knowledge from coursework and nutrition professionals, while male student-athletes indicated strength and conditioning specialists and athletic trainers were their primary sources. Both sexes still relied on friends, family, and magazines [11]. In the 185 student-athletes out of the 579 participants studied by Torres-McGehee et al. they found only 9% of the student-athletes had adequate sports nutrition knowledge on their questionnaire with a score > 75%. For their primary and secondary source of sports nutrition knowledge, the student-athletes reported strength and conditioning specialists to be the highest and then parents and athletic trainers, but not Registered Dietitians [6].

In looking at sports nutrition knowledge in specific teams, Hornstrom et al. studied nutrition knowledge, choices, and practices in 185 collegiate conference softball players [12]. The sports nutrition knowledge mean score on their questionnaire was only 45.7 (4.7) out of 80 points or 57.1%. There were no differences in sports nutrition knowledge by class level. The lower the sports nutrition knowledge scores were, the more the athletes reported poorer eating behaviors. The softball players were most likely to contact a physician to obtain nutrition knowledge, followed by an athletic trainer, college courses, and then a Dietitian [12]. Zawila et al. assessed sports nutrition knowledge and attitudes of 60 female collegiate cross-country runners in two Midwest states. Questions covered topic areas of carbohydrates, protein, fats, calcium, iron, vitamins, minerals, functional foods, vegetables, health benefits of foods, hydration, nutrition for athletes and weight loss, and the runner's attitudes towards nutrition. The runners scored higher than 70% on iron, hydration, and functional foods. Student-athletes who took a nutrition course scored higher, and those who prepared their own food also scored higher. There were no differences between Division I, II, or III universities or between the two states. Sources of sports nutrition knowledge were magazines, parents, coaches, and teammates with only 17% choosing an athletic trainer [13].

It is possible for student-athletes to improve their nutrition knowledge and behavior, although the linking of both of these concepts is complex, and the relationship is somewhat weak at *r* = 0.44 [8]. There are influences from the coach, family, friends, Internet, culture, and religion, as well as issues with study design, participants, comparison populations or lack thereof, and instrumentation [8]. Nascimento et al. found that providing four individualized counseling sessions lasting 45–60 minutes combined with one lecture over an 8-month period improved knowledge, behaviors, and lean body mass in adults and adolescent athletes [10]. There also exists the potential for face-to-face or online coursework that could be offered to improve student-athletes' sports nutrition knowledge and behavior [3, 4].

Optimal nutrition facilitates and enhances physical activity, athletic performance, and recovery [5]. Student-athletes need to understand basic sports nutrition concepts in order to maintain body weight and health while also maximizing training effects. Gaining this knowledge needs to be balanced with their practice and competition schedule, academic coursework, personal preferences, cultural and religious influences, and motivation for behavior change [8]. However, the first step could be basic nutrition knowledge and then followed by behaviors in order to set up some sort of counseling or educational program. The purpose of this study was to determine the sports nutrition knowledge of student-athletes at a mid-major Division I university across sex, class level, team, and prior nutrition coursework.

## 2. Materials and Methods

### 2.1. Participants

The participants for this study were 123 student-athletes from a mid-major Division I university in the southeastern United States. All participants ages 18 and older were asked to volunteer for the study. Predetermined groups based on sport, class level, and sex were generated for convenience. A total of 10 individual and team sports were asked to participate in the study. Coaches were contacted from men's and women's soccer, men's and women's basketball, men's and women's track and field, men's and women's tennis, baseball, and softball. The university does not have a football team. Out of the 10 teams contacted, 7 coaches agreed to participation from baseball, men's and women's tennis, men's and women's track and field, men's soccer, and softball. The participants did not have access to a Registered Dietitian or board certified Sports Dietitian.

### 2.2. Instrument

The sports nutrition knowledge questionnaire was developed by Torres-McGehee et al. [6]. They examined the sports nutrition knowledge and sources of knowledge from 579 student-athletes and coaching staff members among Division I, II, and III universities. Twelve professionals established the construct validity by providing feedback on the questions with respect to their fields of study. Initially the instrument included 50 questions but was revised based on recommendations from the panel. A pilot study was conducted on student-athletes and coaching staff members to obtain additional feedback before they administered the question to the larger sample [6].

In the present study, student-athletes were asked to answer a 19-item questionnaire. Three questions pertained to the student-athlete's demographic information: sex, class rank, and sport. The remaining 16 sports nutrition questions were completed using multiple choice answers including prior nutrition coursework. Examples of questions included “the recommended guideline for safe and healthy weight loss is:” and “from a sports performance perspective, which is the most significant and/or detrimental dietary deficiency?” The sports nutrition question responses were graded to determine the student-athlete's sports nutrition knowledge score. Adequate sports nutrition knowledge was obtained when the student-athletes achieved an overall score of at least 75% out of 100%. An inadequate nutrition knowledge score was below 75% [6].

### 2.3. Data Collection

Permission was given by the athletic department and the head coaches prior to beginning this study. The University's Institutional Review Board (IRB) granted approval to conduct the study. All data were collected in-person at team practices or meetings. The student-athletes were read a script and given a letter explaining the purpose of the study and requesting voluntary participation. The participants were informed about the option to skip questions or leave the study at any time without penalty. Student-athletes completed informed consent forms and then were given the surveys in hard copy. No coaches were in the room at the time of data collection. All survey data were anonymous, and the consent forms were stored separately. Data were collected during the 2013-2014 academic year.

### 2.4. Data Analysis

All data were entered into and analyzed by SPSS Version 22.0 (IBM Corporation, Armonk, NY, 2013). Cronbach's alpha was calculated at 0.92, which may indicate redundancy of items [14]. There was no Cronbach alpha statistic reported in the original study [6]. Independent *t*-tests were used to calculate differences in sports nutrition knowledge scores between males and females, upperclassmen (juniors and seniors) and lowerclassmen (freshmen and sophomores), and completion of a nutrition course. To test differences in scores between class rank and teams, a one-way ANOVA was utilized.

## 3. Results

### 3.1. Overall Sports Nutrition Knowledge Scores

Adequate sports nutrition knowledge was obtained when the student-athletes achieved an overall score of at least 75% out of 100%. An inadequate sports nutrition knowledge score was below 75% [6]. The overall mean sports nutrition knowledge score for all student-athletes was 56.9% (14.3). Only 12 student-athletes achieved an adequate sports nutrition knowledge score of 75% or higher with a mean score of 82% (5.0). The 111 student-athletes who obtained an inadequate sports nutrition knowledge score had a mean score of 54% (12.1).

### 3.2. Sports Nutrition Knowledge between Males and Females

An independent *t*-test was used to determine the mean scores of the female student-athletes (*n* = 47) and male student-athletes (*n* = 76). Neither sex achieved adequate sports nutrition knowledge scores with female student-athletes scoring 56.5 (13.2) and the male student-athletes scoring 57.1 (14.9). No difference was found between male and female student-athletes' sports nutrition knowledge scores: *t*(121) = 0.243 and *P* = 0.446.

### 3.3. Sports Nutrition Knowledge between Upperclassmen and Lowerclassmen

In order to compare the sports nutrition knowledge by class rank and by upperclassmen versus lowerclassmen (*n* = 73), ANOVA and independent *t*-test were used. There were no differences by each class rank (*F*_(4,118)_ = 0.062, *P* = 0.993). When divided into upperclassmen (juniors and seniors, *n* = 50) and lowerclassmen (freshmen and sophomores, *n* = 73) mean scores still were not different at 56.9 (14.8) and 56.8 (14.0), respectively, on the sports nutrition knowledge questionnaire (*t*(121) = 0.061, *P* = 0.952). Both class groups had inadequate sports nutrition knowledge scores.

### 3.4. Sports Nutrition Knowledge between Sport Teams

A one-way ANOVA was used to compare the mean scores of the sports nutrition questionnaire between sport teams, but no significance was found (*F*_(4,118)_ = 0.414, *P* = 0.798). See Table 1 for each team's mean scores. All teams had inadequate sports nutrition knowledge.

### 3.5. Sports Nutrition Knowledge Based on Prior Nutrition Course

When comparing the student-athlete sports nutrition knowledge between those who had previously taken a nutrition course (*n* = 59) and those who had never taken a nutrition course (*n* = 64), the means were not different, 55.6% (15.0) compared to 58.1% (13.4) respectively, *t*(121) = −0.976, and *P* = 0.334. Both groups had inadequate sports nutrition knowledge.

## 4. Discussion

The vast majority of student-athletes had inadequate sports nutrition knowledge scores. Only 12 student-athletes out of 123 participants scored above 75% on the sports nutrition questionnaire. The overwhelming absence of sports nutrition knowledge appears to reflect the lack emphasis on nutrition from the coaches, strength and conditioning specialists, and athletic trainers within the athletics department. Based on previous research by Torres-McGehee et al. the strength and conditioning specialists and athletic trainers had adequate sports nutrition knowledge with scores of 81.6% and 77.8%, respectively, and were higher than the team coaches (65.9%) [6]. The student-athletes scores (54.9%) in their study were comparable to the student-athletes in the current study at 56.9%. On the other hand, Rockwell et al. found that coaches, strength and conditioning specialists, and athletic trainers did not demonstrate high sports nutrition knowledge on their survey [7]. Torres-McGehee et al. concluded that coaches, strength and conditioning specialists, and athletic trainers could include sports nutrition in their continuing education, but to defer to Registered Dietitians for areas that are beyond their scope of practice [6]. Although the sports nutrition knowledge of the coaching staff was not determined in the current study and could be explored in future research, it is likely that sports nutrition knowledge is not being conveyed to the student-athletes. There was no Registered Dietitian or Sports Dietitian available to the student-athletes, even on a consulting basis. Torres-McGehee et al. reported 58.2% of their sample had some access to a Registered Dietitian, but only about half of these were full or part-time staff within their athletics department. The rest were available through student health services or private off-campus practitioners [6]. Contracting the services of a registered Sports Dietitian in the athletic department would take the responsibility of teaching sports nutrition to the student-athletes off the other members of the coaching, strength and conditioning, and athletic training staff so they can focus on sport-specific practice, conditioning, and injuries. The Dietitian could perform individualized or small group counseling or help develop nutrition courses specific for the athletes.

Taking a nutrition course did not affect sports nutrition knowledge scores in the current study. It is possible the student-athletes did not retain the information they were taught, or they do not have the motivation or encouragement to focus on the nutrition component of their own sport and performance. Zawila et al. found in their sample of female distance runners that those participants who took a course scored higher on nutrition knowledge [13]. Distance running is a sport where body weight and nutrition are likely more of a focus, so athletes may be more motivated to increase their sports nutrition knowledge. Nascimento et al. also found that individual consultations combined with a lecture improved athletes' nutrition knowledge along with their nutrition [10]. There is potential to deliver tailored for-credit courses to athletes either face-to-face in a classroom setting or in an online format. However, the additional time burden to athletes would need to be considered [3, 4].

The upperclassmen (*n* = 50) also failed to score higher on the sports nutrition knowledge questionnaire. Research conducted by Hornstrom et al. found similar results when comparing nutrition knowledge scores of softball players with no differences in scores between class years [12]. The years of experience in a college setting do not seem to have an effect on the amount of sports nutrition knowledge in student-athletes. If a student-athlete is not particularly interested and motivated to improve nutrition, any knowledge gained may not be sustained through the collegiate competition years [3]. Furthermore, it is challenging to make associations with previous studies due to inconsistencies in sample sizes, lack of comparison or control groups, and inconsistent instrumentation and interventions [8].

## 5. Conclusions

These study findings open up an area for more research to be conducted about the effects of sports nutrition knowledge and from which professionals student-athletes obtain their knowledge. Future research could include student-athletes' knowledge and behaviors in sport-specific recommendations, pre- and postworkout and competition meals, and foods eaten at campus dining, in residence halls, and in social situations since previous studies using these methods have demonstrated improvements in both knowledge and improved nutrition choices [4, 10]. Future research may wish to examine sports nutrition knowledge for specific populations such as sports that require competition at reduced body weight such as wrestling, track and field, gymnastics, or cross-country. Assessing sports nutrition knowledge and how particular athletes focus on proper nutrition could lead to better comparisons across sports within a college or university.

The effort student-athletes apply to sport-specific on-the-field practice or weight room training should be complemented with a quality nutrition program to provide student-athletes with the best opportunity for peak performance. Student-athletes attempting to perform without quality nutrition could potentially be at nutrition risk and at a disadvantage in competition.

Providing student-athletes with nutrition counseling should allow for improved performance and allow them to maintain energy levels and lean body mass during the competition season and off-season conditioning. Although the strength and conditioning and coaching staffs oversee training and sport-specific practice at smaller Division I schools, particularly those without football programs, they should not be the professionals relied upon to address the sports nutrition knowledge lacking in the student-athletes. The addition of a Board Certification Specialist in Sports Dietitian (CSSD) or at a minimum a Registered Dietitian, even on a consulting basis, would be a recommendation to the athletic department. The CCSD can partner with coaches and other athletics and medical staff, and he/she can work directly with athletes on an individual consulting basis and/or in some sort of small group educational setting. Universities may also wish to explore some sort of nutrition education course for student-athletes, which can be delivered in a face-to-face or online format. Nutrition behaviors should be assessed along with nutrition knowledge. More studies are needed regarding sports nutrition intervention with student-athletes.

## Figures and Tables

**Table 1:** Sports nutrition knowledge questionnaire mean scores by sport team.

Sport	*n*	Mean (SD)
Baseball	22	55.2 (15.0)
Tennis	15	56.7 (15.8)
Track and field	42	57.4 (11.3)
Men's soccer	25	59.4 (16.7)
Softball	19	54.4 (15.4)
Total	123	56.9 (14.3)

*Note*. *n* = number of participants; inadequate nutrition knowledge score mean < 75%; *P* = 0.798 between sport teams.
